# Supplementary material for: Kinetochore component function in C. elegans oocytes revealed by 4D tracking of holocentric chromosomes
Source: Nat Commun. 2023 Jul 7;14:4032. doi: 10.1038/s41467-023-39702-z (PMC10329006; doi:10.1038/s41467-023-39702-z)
Supplement: Supplementary file 1 — Supplementary Information [file 41467_2023_39702_MOESM1_ESM.pdf]

SUPPLEMENTARY FIGURE 1

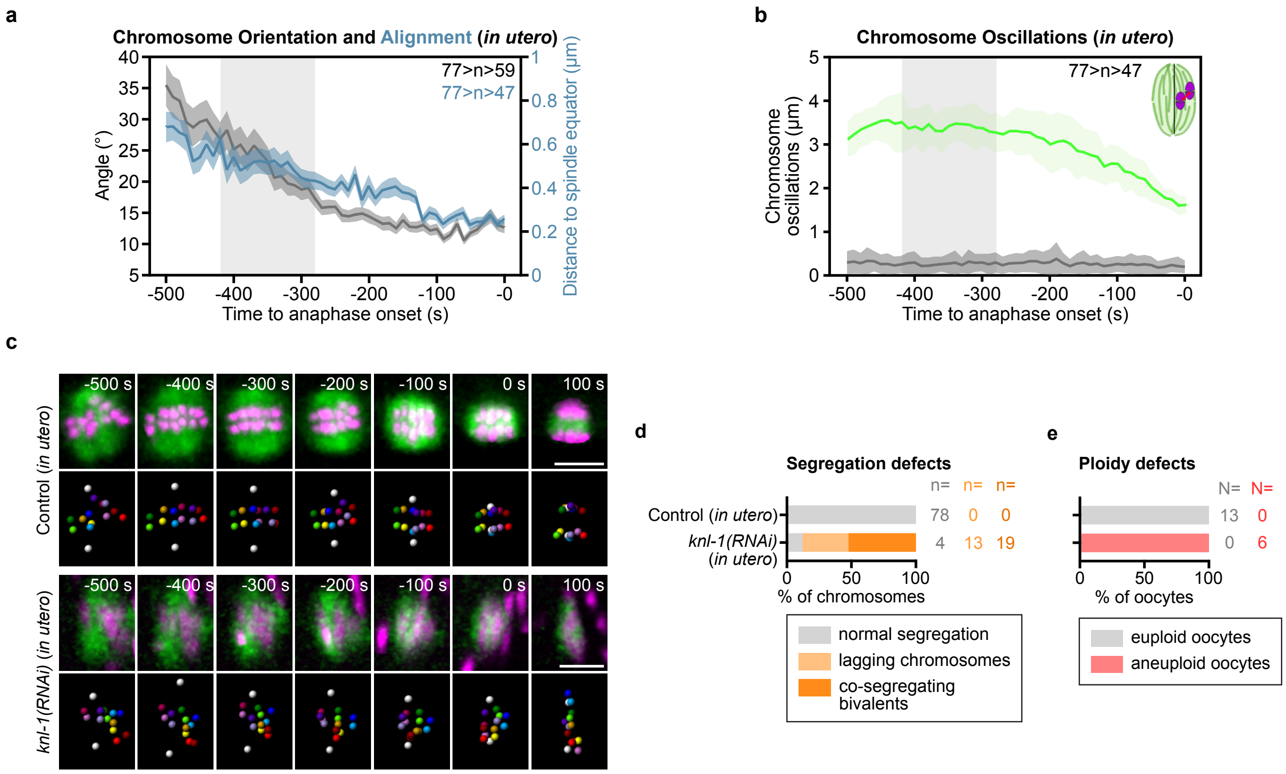

Supplementary Figure 1: Absence of chromosome oscillation along the spindle long axis during their progressive orientation and congression during meiosis I. (a) Plot of mean angle relative to the spindle long axis (grey) and distance to the spindle equator (blue) of the homologous chromosome pairs during the 500 seconds preceding anaphase I onset in oocytes. Dark lines represent the mean. Lighter bands represent the SEM. The grey box highlights the SD of the spindle bipolarization timing centered on the mean timing. Sample size (n pairs of homologous chromosomes) is at the top right of the graph. **(b)** Plot of absolute mean chromosome oscillations (grey) and mean distance of one spindle pole to the spindle equator (green) during the 500 seconds preceding anaphase I onset in oocytes. Dark lines represent the mean. Lighter bands represent the SD. Schematic of the chromosome oscillation analysis and sample size (n pairs of homologous chromosomes) are at the top right of the graph. **(c)** Representative time-lapse images, centered on the meiotic spindle and chromosomes, of GFP::TBA-2$^{a\text{-tubulin}}$ (green) and mCherry::HIS-11^{H2B} (magenta) -expressing oocytes undergoing the first meiotic division in the indicated conditions. Time relative to anaphase onset is indicated at the top right corner of each panel. Scale bars, 5 µm. **(d,e)** Quantification of normally segregating, lagging and co-segregating chromosomes during anaphase (d), and euploid or aneuploid oocytes at the end of anaphase (e) in indicated conditions. Sample sizes (n chromosomes, N oocytes) are at the right of each graph.

SUPPLEMENTARY FIGURE 2

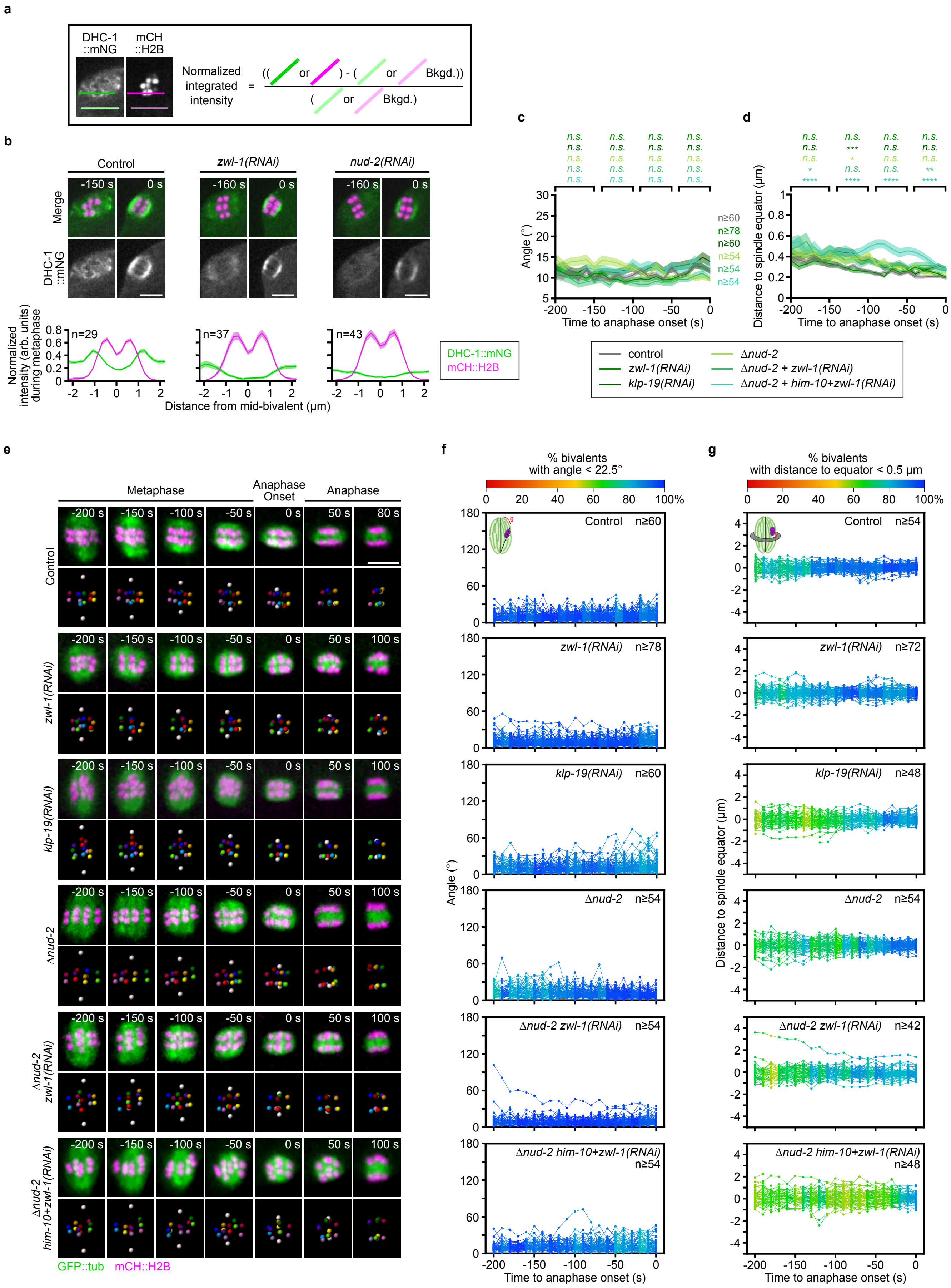

Supplementary Figure 2: NUD-2 and the RZZ complex are both required to recruit dynein at the meiotic cup-like kinetochores. Dynein and the KLP-19 chromokinesin act redundantly to mediate kinetochore-microtubule lateral interactions. (a) Strategy for linescan quantifications of DHC-1^{Dync1h1}::mNeonGreen and mCherry::HIS-11^{H2B} intensities along pairs of homologous chromosomes presented in (b). Colored lines represent linescans along bivalent chromosomes (Green, DHC-1^{Dync1h1}::mNeonGreen ; Magenta, mCherry::HIS-11^{H2B}), light colored lines represent linescans in the corresponding oocyte backgrounds. **(b)** Top: Representative images of DHC-1^{Dync1h1}::mNeonGreen (green) and mCherry::HIS-11^{H2B} (magenta) -expressing oocytes during metaphase (-150 s) and at anaphase onset (0 s) in indicated conditions. Time relative to anaphase onset is indicated at the top right corner of each panel. Scale bars, 5 µm. Bottom: Linescan quantifications of DHC-1^{Dync1h1}::mNeonGreen and mCherry::HIS-11^{H2B} intensities along pairs of homologous chromosomes during anaphase in indicated conditions. Dark lines represent the mean. Lighter bands represent the SEM. Samples sizes (n pairs of homologous chromosomes) is at the top left of each graph. **(c,d)** Plots of mean angle relative to the spindle long axis (c) and distance to the spindle equator (d) of the homologous chromosome pairs during the 200 seconds preceding anaphase I onset in oocytes in the indicated conditions. Dark lines represent the mean. Lighter bands represent the SEM. Samples sizes (n pairs of homologous chromosomes) are at the right of each graph. (one-way ANOVA with Tukey's multiple comparison: n.s., $P \geq 0.05$; **, $P < 0.01$; ***, $P < 0.001$; and ****, $P < 0.0001$). **(e)** Representative time-lapse images, centered on the meiotic spindle and chromosomes, of GFP::TBA-2$^{\alpha\text{-tubulin}}$ (green) and mCherry::HIS-11^{H2B} (magenta) -expressing oocytes during end of metaphase and anaphase in the indicated conditions. Time relative to anaphase onset is indicated at the top right corner of each panel. Scale bars, 5 µm. **(f,g)** Plots of individual homologous chromosome pair angles relative to the spindle long axis

(f) or of the distance between individual homologous chromosome pairs and the spindle equator (g) during the 200 seconds preceding anaphase I onset in oocytes, in the indicated conditions. Each dot corresponds to an individual homologous chromosome pair and is color-coded as indicated at the top of each graph. Schematics of the measured parameter and sample size (n pairs of homologous chromosomes) are at the top left and right corners respectively of each graph.

SUPPLEMENTARY FIGURE 3

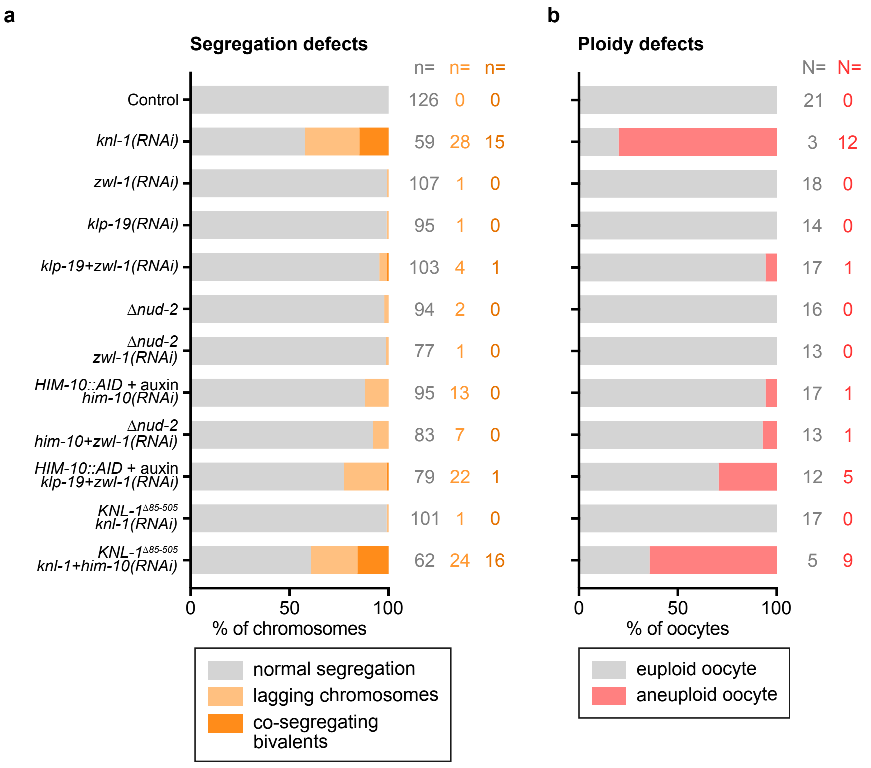

Supplementary Figure 3: The only perturbation tested that recapitulates KNL-1-depleted oocytes mis-segregating phenotype is the absence of both end-on connections and kinetochore CLS-2. (a,b) Quantification of normally segregating, lagging and co-segregating chromosomes during anaphase (a), and euploid or aneuploid oocytes at the end of anaphase (b) in indicated conditions. Sample sizes (n chromosomes, N oocytes) are at the right of each graph.

SUPPLEMENTARY FIGURE 4

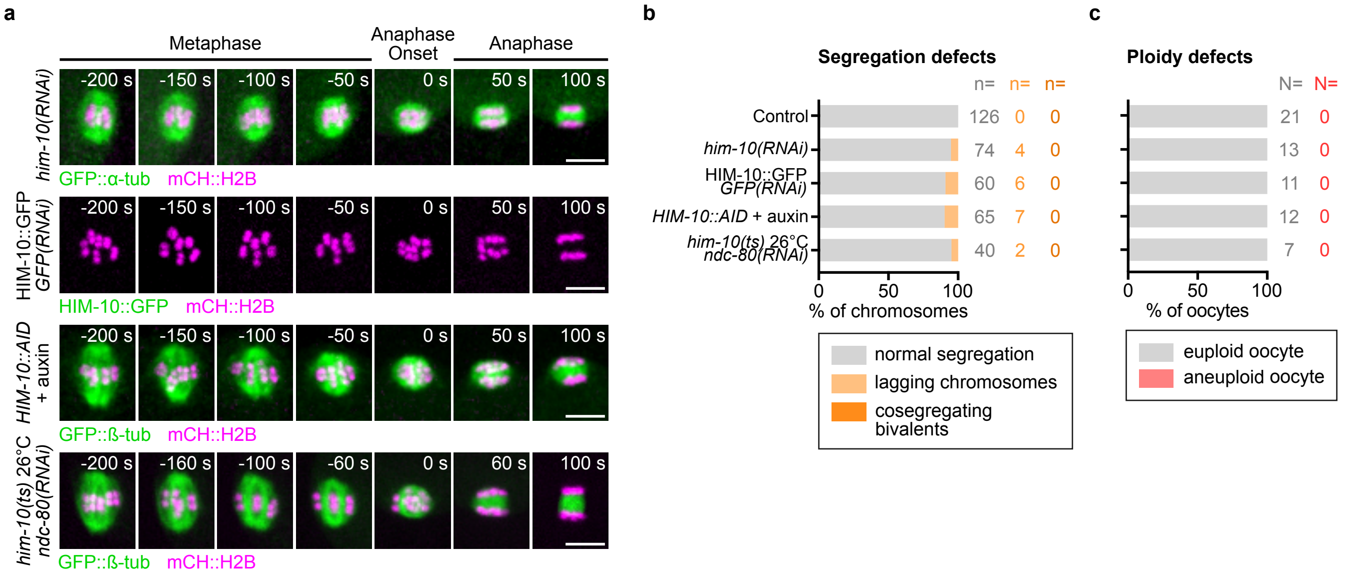

Supplementary Figure 4: Ndc80 complex-mediated end-on connections alone are not essential for chromosome segregation. (a) Representative time-lapse images, centered on the meiotic spindle and chromosomes, of GFP::TBA-2$^{\alpha\text{-tubulin}}$ or GFP::TBB-2$^{\beta\text{-tubulin}}$ or HIM-10^{Nuf2}::GFP (green) and mCherry::HIS-11^{H2B} (magenta) -expressing oocytes during end of metaphase and anaphase in the indicated conditions. Time relative to anaphase onset is indicated at the top right corner of each panel. Scale bars, 5 µm. **(b,c)** Quantification of normally segregating, lagging and co-segregating chromosomes during anaphase (b), and euploid or aneuploid oocytes at the end of anaphase (c) in indicated conditions. Sample sizes (n chromosomes, N oocytes) are at the right of each graph.

SUPPLEMENTARY FIGURE 5

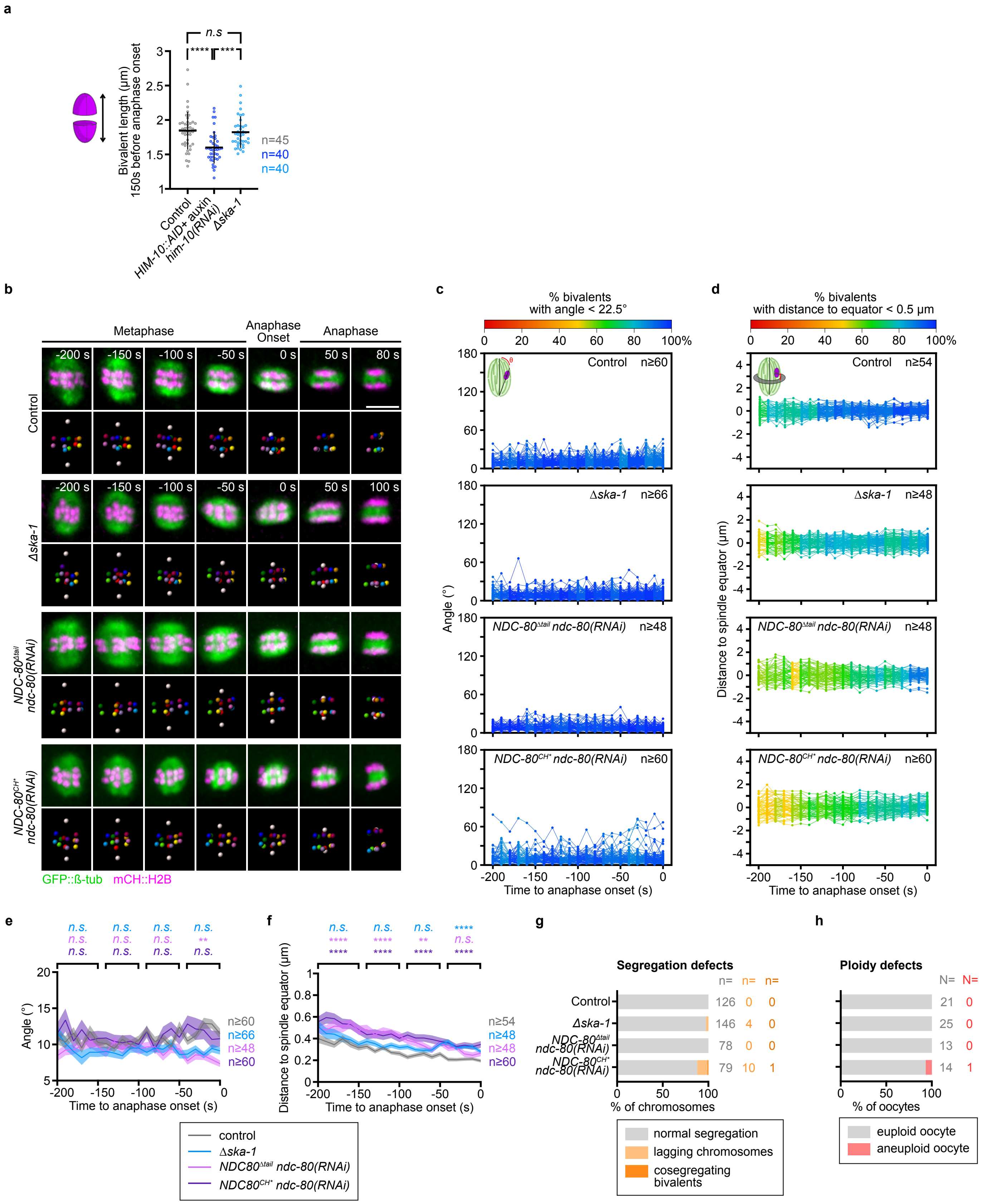

Supplementary Figure 5: Involvement of the NDC-80 tail and CH domain, and of the SKA complex in chromosome alignment. (a) Plot of bivalent chromosome length 150 seconds before anaphase onset in the indicated conditions. Errors bars represent the SD. Samples size (n pairs of homologous chromosomes) are at the right of the graph. (one-way ANOVA with Tukey's multiple comparison: n.s., P≥0.05; **, P < 0.01; ***, P < 0.001; and ****, P < 0.0001). **(b)** Representative time-lapse images, centered on the meiotic spindle and chromosomes, of GFP::TBA-2$^{\alpha\text{-tubulin}}$ (green) and mCherry::HIS-11^{H2B} (magenta) -expressing oocytes during end of metaphase and anaphase in the indicated conditions. Time relative to anaphase onset is indicated at the top right corner of each panel. Scale bars, 5 µm. **(c,d)** Plots of individual homologous chromosome pair angles relative to the spindle long axis (c) or of the distance between individual homologous chromosome pairs and the spindle equator (d) during the 200 seconds preceding anaphase I onset in oocytes, in the indicated conditions. Each dot corresponds to an individual homologous chromosome pair and is color-coded as indicated at the top of each graph. Schematics of the measured parameter and sample size (n pairs of homologous chromosomes) are at the top left and right corners respectively of each graph. **(e,f)** Plots of mean angle relative to the spindle long axis (e) and distance to the spindle equator (f) of the homologous chromosome pairs during the 200 seconds preceding anaphase I onset in oocytes, in the indicated conditions. Dark lines represent the mean. Lighter bands represent the SEM. Samples sizes (n pairs of homologous chromosomes) are at the right of each graph. (one-way ANOVA with Tukey's multiple comparison: n.s., P≥0.05; **, P < 0.01; ***, P < 0.001; and ****, P < 0.0001). **(g,h)** Quantification of normally segregating, lagging and co-segregating chromosomes during anaphase (g), and euploid or aneuploid oocytes at the end of anaphase (h) in indicated conditions. Sample sizes (n chromosomes, N oocytes) are at the right of each graph.

SUPPLEMENTARY FIGURE 6

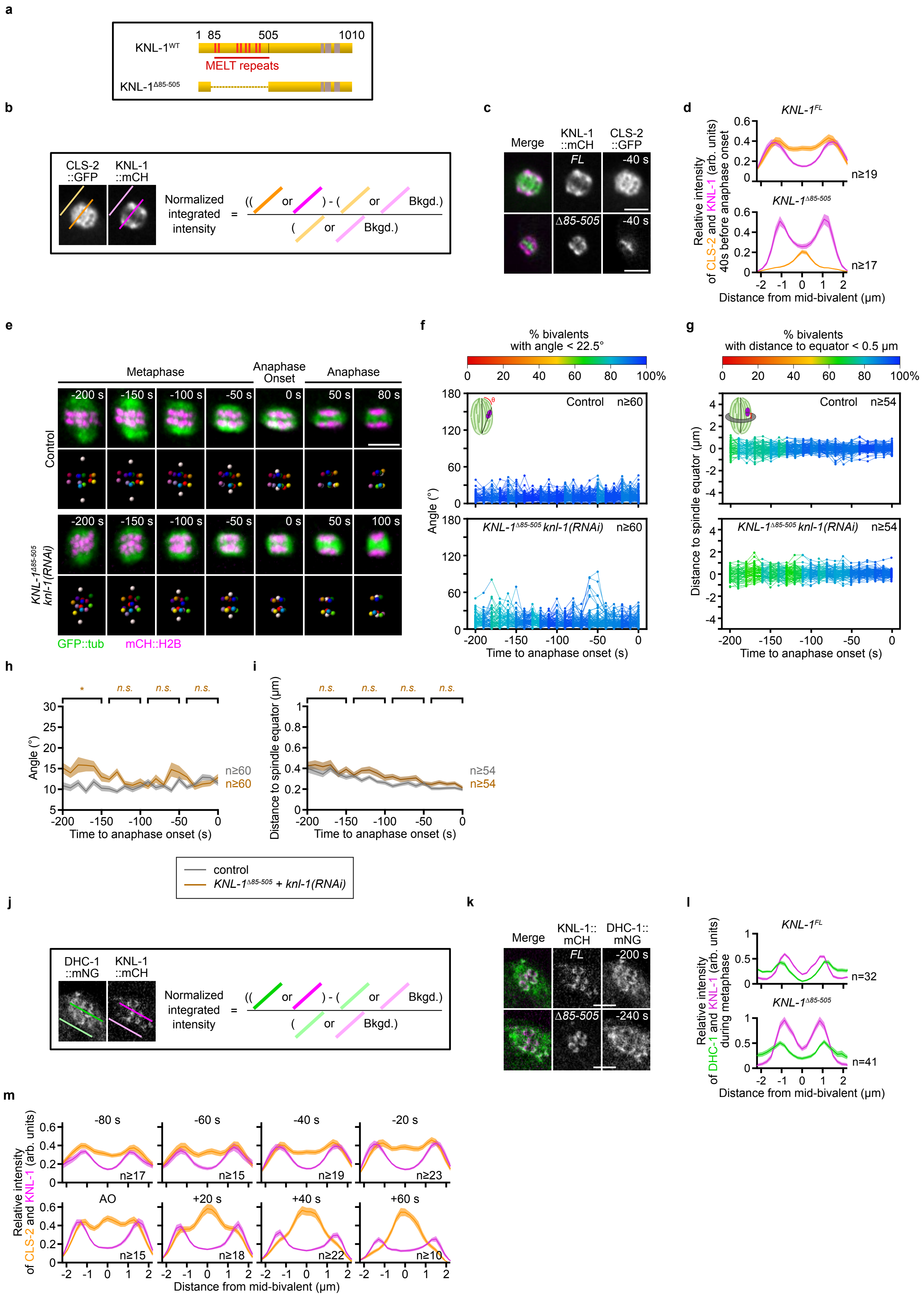

Supplementary Figure 6: Kinetochore-localized CLS-2 alone is not essential for accurate chromosome segregation. (a) Schematics of wild-type (WT) KNL-1 and of the MELTs-deletion mutant (KNL-1 *Δ85-505*). **(b)** Strategy for linescan quantification of CLS-2^{Clasp2}::GFP and KNL-1^{Spc105}::mCherry intensities along pairs of homologous chromosomes presented in (d). **(c)** Representative images, centered on the meiotic spindle and chromosomes, of CLS-2^{Clasp2}::GFP (green) and KNL-1^{Spc105}::mCherry (magenta) -expressing oocytes 40 s before anaphase onset in the indicated conditions. Time relative to anaphase onset is indicated on the top right corner of each panel. Scale bars, 5 µm. **(d)** Linescan quantifications of CLS-2^{Clasp2}::GFP and KNL-1^{Spc105}::mCherry intensities along pairs of homologous chromosomes 40 s before anaphase onset. Dark lines, mean; lighter bands, SEM. Sample sizes (n pairs of homologous chromosomes) at the bottom right corner of each graph. **(e)** Representative time-lapse images, centered on the meiotic spindle and chromosomes, of GFP::TBA-2$^{\alpha\text{-tubulin}}$ or GFP::TBB-2$^{\beta\text{-tubulin}}$ (green) and mCherry::HIS-11^{H2B} (magenta) -expressing oocytes during end of metaphase and anaphase in the indicated conditions. Time relative to anaphase onset is indicated at the top right corner of each panel. Scale bars, 5 µm. **(f,g)** Plots of individual homologous chromosome pair angles relative to the spindle long axis (f) or of the distance between individual homologous chromosome pairs and the spindle equator (g) during 200 seconds preceding anaphase onset in oocytes, in the indicated conditions. Color-code is indicated at the top of each graph. Schematics of the measured parameter and sample size (n pairs of homologous chromosomes) are at the top left and right corners respectively of each graph. **(h,i)** Plots of mean angle relative to the spindle long axis (h) and distance to the spindle equator (i) of the homologous chromosome pairs during 200 seconds preceding anaphase onset in oocytes, in the indicated conditions. Dark lines, mean; lighter bands, SEM. Samples sizes (n pairs of homologous chromosomes) are at the right of each graph. (one-way ANOVA with Tukey's

multiple comparison: n.s., P≥0.05; **, P < 0.01; ***, P < 0.001; and ****, P < 0.0001). **(j)** Strategy for linescan quantification of DHC-1^{Dync1h1}::mNeonGreen and KNL-1^{Spc105}::mCherry intensities along pairs of homologous chromosomes presented in (l). **(k)** Representative images, centered on the meiotic spindle and chromosomes, of DHC-1^{Dync1h1}::mNeonGreen (green) and KNL-1^{Spc105}::mCherry (magenta) -expressing oocytes 200 s or 240 s before anaphase onset in the indicated conditions. Time relative to anaphase onset is indicated on the top right corner of each panel. Scale bars, 5 µm. **(l)** Linescan quantifications of DHC-1^{Dync1h1}::mNeonGreen and KNL-1^{Spc105}::mCherry intensities along pairs of homologous chromosome during metaphase. Dark lines, mean; Lighter bands, SEM. Samples sizes (n pairs of homologous chromosomes) is at the bottom right corner of each graph. **(m)** Linescan quantifications of CLS-2^{Clasp2}::GFP and KNL-1^{Spc105}::mCherry intensities along pairs of homologous chromosomes from metaphase to early anaphase. Time relative to anaphase onset is at the top of each graph. Dark lines, mean; Lighter bands, SEM. Sample sizes (n pairs of homologous chromosomes) is at the bottom right corner of each graph.

SUPPLEMENTARY FIGURE 7

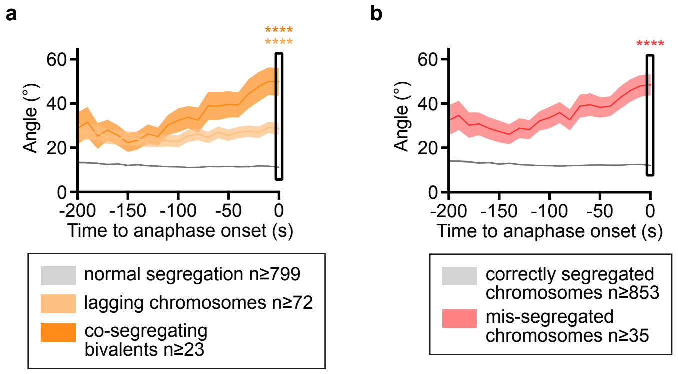

Supplementary Figure 7: Correlation between chromosome orientation during metaphase and the segregation output. (a) Mean angle of homologous chromosome pairs relative to the spindle long axis over time for normally segregating (grey), lagging (light orange) and co-segregating (dark orange) chromosomes in oocytes for all conditions analyzed in 3D in this study. Sample sizes (n chromosomes) are in the key. Dark lines represent the mean. Lighter bands represent the SEM. (one-way ANOVA with Tukey's multiple comparison: n.s., $P \geq 0.05$; **, $P < 0.01$; ***, $P < 0.001$; and ****, $P < 0.0001$). **(b)** Mean angle of homologous chromosome pairs relative to the spindle long axis over time for correctly segregated (grey) and mis-segregated (red) chromosomes in oocytes for all conditions analyzed in 3D in this study. Sample sizes (n chromosomes) are in the key. Dark lines represent the mean. Lighter bands represent the SEM. (t-tests with Welch's correction, two-sided: n.s., $P \geq 0.05$; **, $P < 0.01$; ***, $P < 0.001$; and ****, $P < 0.0001$).

SUPPLEMENTARY TABLES

Supplementary Table 1. *C. elegans* strains used

Strain	Genotype
N2	*Wild Type (Ancestral N2 Bristol)*
JDU233	*ijmSi63 [pJD520; mosII_5'mex-5_gfp::tba-2; mCherry::his-11; cb-unc-119(+)] II; unc-119(ed3) III?*
JDU19	*ijmSi7 [pJD348; Pmex-5_gfp::tbb-2; mCherry::his-11; cb-unc-119(+)] I; unc-119(ed3) III.*
JDU659	*ijmSi7 [pJD348; Pmex-5_gfp::tbb-2; mCherry::his-11; cb-unc-119(+)] I; unc-119(ed3) III ?; wrdSi3 [sun-1p::TIR1::F2A::BFP::AID*::NLS::tbb-2 3'UTR] II:-0.77; him-10(syb3493[him-10::mAID])III*
JDU564	*ska-1(lt29[loxp])I. ijmSi7 [pJD348; Pmex-5_gfp::tbb-2; mCherry::his-11; cb-unc-119(+)] I; unc-119(ed3) III?*
JDU677	*ijmSi7 [pJD348; Pmex-5_gfp::tbb-2; mCherry::his-11; cb-unc-119(+)] I; unc-119(ed3) III; ltSi121[pDC175;Pndc-80:NDC-80 (Mutant D1-59) reencoded; cb-unc- 119(+)]II; unc-119(ed3)III*
JDU678	*ijmSi7 [pJD348; Pmex-5_gfp::tbb-2; mCherry::his-11; cb-unc-119(+)] I; ltSi711[pDC267;Pndc-80:NDC80(66,96,100,125,144,155AAAAAA) reencoded; cb-unc-119(+) II#1; unc-119(ed3)III ?*
JDU321	*ijmSi7 [pJD348/pSW077; mosI_5'mex-5_GFP::tbb-2; mCherry::his-11; cb-unc-119(+)]I; ltSi1[pOD809/pJE110; Pknl-1::KNL-1reencoded::mCherry; cb-unc-119(+)]II; unc-119(ed3) III?*
JDU323	*ijmSi7 [pJD348/pSW077; mosI_5'mex-5_gfp::tbb-2; mCherry::his-11; cb-unc-119(+)]I; ltSi44[pOD1039/pJE170; Pknl-1::KNL-1reencoded(Mutant D85-505)::mCherry; cb-unc-119(+)]II; unc-119(ed3) III?*
JDU31	*unc-119(ed3) III; ltSi44[pOD1039/pJE170; Pknl-1::KNL-1reencoded(Mutant D85-505)::mCherry; cb-unc-119(+)]II; unc-119(ed3) III; ijmSi3 [pJD342/pJD330; ChrI_5'mex-5_cls-2reenc::gfp_tbb-2; cb-unc-119(+)]I*
JDU35	*unc-119(ed3) III; ltSi1[pOD809/pJE110; Pknl-1::KNL-1reencoded::mCherry; cb-unc-119(+)]II; unc-119(ed3) III; ijmSi3 [pJD342/pJD330; ChrI_5'mex-5_cls-2reenc::GFP_tbb-2; cb-unc-119(+)]I*
JDU667	*dhc-1(cp268[dhc::mNG-C1^3xFlag]) I.; ijmSi31 [pJD446_pJD362_Mos2_Pmex-5_mCherry_his11_3'UTRtbb-2]II; unc-119(ed3) III?*
JDU546	*ijmSi31 [pJD446_pJD362_Mos2_Pmex-5_mCherry::his-11_3'UTRtbb-2] II; unc-119(ed3) III?; him-10(lt52[him-10::sGFP) III.*
JDU530	*lt28[ska-1::gfp + Loxp]I; ijmSi31 [pJD446_pJD362_Mos2_Pmex-5_mCherry::his-11_3'UTRtbb-2] II*
JCC483	*ojls1[Ppie-1_gfp::tbb-2]; unc-119(ed3) III?; ltIs37[pAA64; Ppie-1_mCherry::his-58; unc-119 (+)] IV.*
JDU574	*nud-2(ok949) I. ijmSi63 [pJD520; mosII_5'mex-5_gfp::tba-2; mCherry::his-11; cb-unc-119(+)] II; unc-119(ed3) III?*
JDU537	*ijmSi7 [pJD348; Pmex-5_gfp::tbb-2; mCherry::his-11; cb-unc-119(+)] I; unc-119(ed3)?, him-10(e1511)*ts *III.*
JDU780	*dhc-1(cp268[dhc::mNG-C1^3xFlag]) I; ltSi1[pOD809/pJE110; Pknl-1::KNL-1reencoded::mCherry; cb-unc-119(+)] II; unc-119(ed3) III?*
JDU782	*dhc-1(cp268[dhc::mNG-C1^3xFlag]) I; ltSi44[pOD1039/pJE170; Pknl-1::KNL-1reencoded(Mutant D85-505)::mCherry; cb-unc-119(+)]II; unc-119(ed3) III?*
PHX3493	*him-10(syb3493[him-10::mAID])III*
JDW10	*wrdSi3 [sun-1p::TIR1::F2A::BFP::AID*::NLS::tbb-2 3'UTR] II:-0.77*

*[*unc-119(ed3)?*] was present in the parental strains, but these strains have not been directly sequenced to determine if the *unc-119* gene contains the *ed3* mutation.

Supplementary Table 2. Templates and primers used for *dsRNA* production

Gene	Primer 1*	Primer 2*	Template	Final [C] (µg/µl)	Ref. for depletion efficiency
C02F5.1 *(knl-1)*	5'-aattaaccctcactaaagg AATCTCGAATCACCGAAATGTC-3'	5'-taatacgactcactatagg TTCACAAACTTGGAAGCCGCTG-3'	N2 genomic DNA	1,6	1
Y39G10AR.2 *(zwl-1)*	5'-aattaaccctcactaaagg ATGCCACTCACCATCGAGCAG-3'	5'taatacgactcactatagg GGATCAGTGAAGCGAGATGACTC-3'	N2 cDNA	1,3	2
Y43F4B.6 *(klp-19)*	5'-aattaaccctcactaaagg GCGAAGCACGTAGGAAAGTC-3'	5'-taatacgactcactatagg TGATGCAGCTGAAGTGGTTC-3'	N2 genomic DNA	1,8	3
R12B2.4 *(him-10)*	5'-aattaaccctcactaaagg ATTCTGGCAACAAGCTGGAC-3'	5'-taatacgactcactatagg ACGCTGACGCTCTTCACTTT-3'	N2 genomic DNA	1,6	4
W01B6.9 *(ndc-80)*	5'-aattaaccctcactaaagg GATGACAAGTACATTCAGAGATTATACAAA TGATC-3'	5'-taatacgactcactatagg GTGGTTCAAGATTCATTTGAATATTAAGTCCA CTG-3'	N2 cDNA	1,3	5
R11A5.2 *(nud-2)*	5'-aattaaccctcactaaagg ATGGATTTGTCTGAGGATCAAATTCG-3'	5'-taatacgactcactatagg GATGACTGGAATTGTTGTTAGACG-3'	N2 cDNA	1,1	No antibody available
GFP	5'-aattaaccctcactaaagg TCCACCTCCTCCTCCTCC-3'	5'-taatacgactcactatagg TTCACTGGAGTTGTCCC-3'	Plasmid pJD477	1,2	This study

*Lowercase letters denote T3 and T7 sequences included for RNA synthesis

Supplementary Table 3. Images and datasets presented in different figures and panels

Condition	Fluorescence images	Chromosome orientation	Chromosome alignment	Chromosome axial compaction	Segregation defects	Ploidy defects	Angles relative to segregation	Angles relative to ploidy	Mean chromosome orientation	Mean chromosome alignment	Homolog length	Inter-homolog distance	Bivalent length	Fluorescence intensity measurement	Chromosome oscillations
JDU233 Control *in utero*	F1b, F1c, F1d	F1e	F1f	F1g					SF1a	SF1a					SF1b
JDU233 *knl-1(RNAi) in-utero*	SF1c				SF1d	SF1e									
JDU233 *knl-1(RNAi) ex utero*	F2a, F2b, F2c	F2d	F2e		F2f, F3f, SF3a	F2g, F3g, SF3b	F2h, SF7a	F2i, SF7b	F3d	F3e					
JDU233 Control *ex utero*	F2c, F3a, F4d, SF2e, SF5b, SF6e	F2d, F3b, SF2f, SF5c, SF6f	F2e, F3c, SF2g, SF5d, SF6g		F2f, F3f, SF3a, SF5g	F2g, F3g, SF3b, SF5h	SF7a	SF7b	F3d, SF2c, SF5e, SF6h	F3e, SF2d, SF5f, SdF6i	F4a	F4b, F5f	F4c, F4d, F5e		
JDU233 *klp-19+zwl-1(RNAi)*	F3a	F3b	F3c		F3f, SF3a	F3g, SF3b	SF7a	SF7b	F3d	F3e					
JDU659 Aux *him-10(RNAi)*	F3a, F4d	F3b	F3c		F3f, SF3a	F3g, SF3b	SF7a	SF7b	F3d	F3e		F5f	F4d, F4h, F5e, SF5a		
JDU659 Aux *klp-19+zwl-1(RNAi)*	F3a	F3b	F3c		F3f, SF3a	F3g, SF3b	SF7a	SF7b	F3d	F3e					
JDU323 *knl-1+him-10(RNAi)*	F3a	F3b	F3c		F3f, SF3a	F3g, SF3b	SF7a	SF7b	F3d	F3e		F5f	F5e		
JCC483 Control + nocodazole	F4e													F4f	
JCC483 him-10(RNAi) + nocodazole	F4e													F4f	
JDU574 zwl-1(RNAi) + nocodazole	F4e													F4f	
JDU19 Control													F4h, SF5a		
JDU564	SF5b	SF5c	SF5d		SF5g	SF5h	SF7a	SF7b	SF5e	SF5f			F4h, SF5a		
JDU677 *ndc-80(RNAi)*	SF5b	SF5c	SF5d		SF5g	SF5h	SF7a	SF7b	SF5e	SF5f			F4h		
JDU678 *ndc-80(RNAi)*	SF5b	SF5c	SF5d		SF5g	SF5h	SF7a	SF7b	SF5e	SF5f			F4h		

JDU574 *klp-19+zwl-1(RNAi)*													F4h		
JDU546	F4i													F4k	
JDU530	F4j													F4k	
JDU31	F5b, SF6c,													F5a, SF6d	
JDU35	F5b, SF6b, SF6c,													F5a, SF6d, SF6m	
JDU321	F5d													F5c	
JDU323 4Z	F5d													F5c	
JDU323 knl-1(RNAi)	SF6e	SF6f	SF6g		SF3a	SF3b	SF7a	SF7b	SF6h	SF6i		F5f	F5e		
JDU667	SF2a, SF2b													SF2b	
JDU667 *zwl-1(RNAi)*	SF2b													SF2b	
JDU667 *nud-2(RNAi)*	SF2b													SF2b	
JDU233 *zwl-1(RNAi)*	SF2e	SF2f	SF2g		SF3a	SF3b	SF7a	SF7b	SF2c	SF2d					
JDU233 *klp-19(RNAi)*	SF2e	SF2f	SF2g		SF3a	SF3b	SF7a	SF7b	SF2c	SF2d					
JDU574	SF2e	SF2f	SF2g		SF3a	SF3b	SF7a	SF7b	SF2c	SF2d					
JDU574 *zwl-1(RNAi)*	SF2e	SF2f	SF2g		SF3a	SF3b	SF7a	SF7b	SF2c	SF2d					
JDU574 *him-10+zwl-1(RNAi)*	SF2e	SF2f	SF2g		SF3a	SF3b	SF7a	SF7b	SF2c	SF2d					
JDU233 *him-10(RNAi)*	SF4a				SF4b	SF4c									
JDU546 *GFP(RNAi)*	SF4a				SF4b	SF4c									
JDU659 Aux1mM	SF4a				SF4b	SF4c									
JDU537 26°C *ndc-80(RNAi)*	SF4a				SF4b	SF4c									
JDU780 *knl-1(RNAi)*	SF6j, SF6k													SF6l	
JDU782 *knl-1(RNAi)*	SF6k													SF6l	

Supplementary Table 4. P-values of statistical tests performed in this study

**Fig2h**<br>one-way ANOVA with Tukey's multiple comparison	**Normal segregators vs laggers at anaphase onset**	0,0316<br>*			
	**Normal segregators vs cossegregators at anaphase onset**	<0,0001<br>****			
**Fig2i**<br>t-test with Welch's correction, two-sided	**Correctly vs non correctly segregated chromosomes at anaphase onset**	0,0046<br>**			
**Fig3d**<br>one-way ANOVA with Tukey's multiple comparison (of all 3D conditions)		**-200s to -150s**	**-140s to -100s**	**-90s to -50s**	**-40s to 0s**
	**Control vs *knl-1(RNAi)***	0,0053<br>**	<0,0001<br>****	<0,0001<br>****	<0,0001<br>****
	**Control vs *klp-19+zwl-1(RNAi)***	<0,0001<br>****	0,0003<br>***	0,9997<br>n.s.	>0,9999<br>n.s.
	**Control vs *HIM-10::AID* + auxin + *him-10(RNAi)***	>0,9999<br>n.s.	0,8776<br>n.s.	0,8381<br>n.s.	0,5173<br>n.s.
	**Control vs *HIM-10::AID* + auxin + *klp-19+zwl-1(RNAi)***	<0,0001<br>****	<0,0001<br>****	<0,0001<br>****	<0,0001<br>****
	**Control vs *KNL-1 Δ85-505* + *knl-1+him-10(RNAi)***	<0,0001<br>****	<0,0001<br>****	<0,0001<br>****	<0,0001<br>****
**Fig3e**<br>one-way ANOVA with Tukey's multiple comparison (of all 3D conditions)		**-200s to -150s**	**-140s to -100s**	**-90s to -50s**	**-40s to 0s**
	**Control vs *knl-1((RNAi)***	<0,0001<br>****	<0,0001<br>****	<0,0001<br>****	<0,0001<br>****
	**Control vs *klp-19+zwl-1(RNAi)***	<0,0001<br>****	<0,0001<br>****	0,918<br>n.s.	0,6206<br>n.s.
	**Control vs *HIM-10::AID* + auxin + *him-10(RNAi)***	<0,0001<br>****	<0,0001<br>****	<0,0001<br>****	<0,0001<br>****
	**Control vs *HIM-10::AID* + auxin + *klp-19+zwl-1(RNAi)***	<0,0001<br>****	<0,0001<br>****	<0,0001<br>****	<0,0001<br>****
	**Control vs *KNL-1Δ85-505* + *knl-1+him-10(RNAi)***	<0,0001<br>****	<0,0001<br>****	<0,0001<br>****	<0,0001<br>****
**Fig4d**<br>t-test with Welch's correction, two-sided	**-200s Control vs -200s *HIM-10::AID* + auxin + *him-10(RNAi)***	0,4074<br>n.s.			
	**-200s vs -100s Control**	0,0004<br>***			
	**-200s vs -100s *HIM-10::AID* + auxin + *him-10(RNAi)***	0,0157<br>*			
**Fig4f**<br>one-way ANOVA with Tukey's multiple comparison	**Control vs *him-10(RNAi)***	0,0076<br>**			
	**Control vs *Δnud-2* + *zwl-1(RNAi)***	0,2491<br>n.s.			
**Fig4h**<br>one-way ANOVA with Tukey's multiple comparison	**Control vs *HIM-10::AID* + auxin + *him-10(RNAi)***	<0,0001<br>****			
	**Control vs *Δska-1***	0,0068<br>**			
	**Control vs NDC-80 CH* + *ndc-80(RNAi)***	0,0012<br>**			
	**Control vs NDC-80 *Δtail* + *ndc-80(RNAi)***	0,9574<br>n.s.			

	**Control vs *Δnud-2 + klp-19+zwl-1(RNAi)***	0,1592<br>n.s.
	HIM-10::AID* + auxin + *him-10(RNAi)* vs *Δska-1	0,1334<br>n.s.
	HIM-10::AID* + auxin + *him-10(RNAi)* vs NDC-80 CH* + *ndc-80(RNAi)	0,8012<br>n.s.
	HIM-10::AID* + auxin + *him-10(RNAi)* vs NDC-80 *Δtail* + *ndc-80(RNAi)	<0,0001<br>****
	HIM-10::AID* + auxin + *him-10(RNAi)* vs *Δnud-2 + klp-19+zwl-1(RNAi)	<0,0001<br>****
	Δska-1* vs NDC-80 CH* + *ndc-80(RNAi)	0,9286<br>n.s.
	Δska-1* vs NDC-80 *Δtail* + *ndc-80(RNAi)	<0,0001<br>****
	Δska-1* vs *Δnud-2 + klp-19+zwl-1(RNAi)	<0,0001<br>****
	**NDC-80 CH* + *ndc-80(RNAi)* vs NDC-80 *Δtail* + *ndc-80(RNAi)***	<0,0001<br>****
	**NDC-80 CH* + *ndc-80(RNAi)* vs *Δnud-2 + klp-19+zwl-1(RNAi)***	<0,0001<br>****
	**NDC-80 *Δtail* + *ndc-80(RNAi)* vs *Δnud-2 + klp-19+zwl-1(RNAi)***	0,5297<br>n.s.
**Fig5a**<br>t-test with Welch's correction, two-sided	**-20s KNL-1 WT vs KNL-1 *Δ85-505***	<0,0001<br>****
	**20s KNL-1 WT vs KNL-1 *Δ85-505***	<0,0001<br>****
	**60s KNL-1 WT vs KNL-1 *Δ85-505***	<0,0001<br>****
	**100s KNL-1 WT vs KNL-1 *Δ85-505***	0,0004<br>***
**Fig5c**<br>t-test with Welch's correction, two-sided	**-20s KNL-1 WT vs KNL-1 *Δ85-505***	0,0057<br>**
	**20s KNL-1 WT vs KNL-1 *Δ85-505***	0,0059<br>**
	**60s KNL-1 WT vs KNL-1 *Δ85-505***	0,0022<br>**
	**100s KNL-1 WT vs KNL-1 *Δ85-505***	0,0758<br>n.s.
**Fig5e**<br>one-way ANOVA with Tukey's multiple comparison	**Control vs KNL-1 *Δ85-505* + *knl-1(RNAi)***	0,0051<br>**
	**Control vs *HIM-10::AID* + auxin + *him-10(RNAi)***	<0,0001<br>****
	**Control vs KNL-1 *Δ85-505* + *knl-1+him-10(RNAi)***	<0,0001<br>****
	**KNL-1 *Δ85-505* + *knl-1(RNAi)* vs *HIM-10::AID* +**	<0,0001<br>****

	**auxin + *him-10(RNAi)***				
	**KNL-1 *Δ85-505 + knl-1(RNAi)* vs KNL-1 *Δ85-505 + knl-1+him-10(RNAi)***	<0,0001<br>****			
	HIM-10::AID* + auxin + *him-10(RNAi)* vs KNL-1 *Δ85-505 + knl-1+him-10(RNAi)	0,3979<br>n.s.			
**Fig5f**<br>t-test with Welch's correction, two-sided	**Control -10s to 0s**	<0,0001<br>****			
	**Control 0s to 30s**	<0,0001<br>****			
	**KNL-1 *Δ85-505 + knl-1(RNAi)* -10s to 0s**	<0,0001<br>****			
	**KNL-1 *Δ85-505 + knl-1(RNAi)* 0s to 30s**	0,0005<br>***			
	***HIM-10::AID* + auxin + *him-10(RNAi)* -10s to 0s**	<0,0001<br>****			
	***HIM-10::AID* + auxin + *him-10(RNAi)* 0s to 30s**	<0,0001<br>****			
	**KNL-1 *Δ85-505 + knl-1+him-10(RNAi)* -10s to 0s**	0,0118<br>*			
	**KNL-1 *Δ85-505 + knl-1+him-10(RNAi)* 0s to 30s**	0,051<br>n.s.			
**Sup. Fig2c**<br>one-way ANOVA with Tukey's multiple comparison (of all 3D conditions)		**-200s to -150s**	**-140s to -100s**	**-90s to -50s**	**-40s to 0s**
	**Control to *zwl-1(RNAi)***	>0,9999<br>n.s.	>0,9999<br>n.s.	>0,9999<br>n.s.	>0,9999<br>n.s.
	**Control to *klp-19(RNAi)***	>0,9999<br>n.s.	>0,9999<br>n.s.	>0,9999<br>n.s.	0,9809<br>n.s.
	**Control to *Δnud-2***	0,5251<br>n.s.	0,1797<br>n.s.	>0,9999<br>n.s.	0,9991<br>n.s.
	**Control to *Δnud-2 + zwl-1(RNAi)***	>0,9999<br>n.s.	>0,9999<br>n.s.	0,8596<br>n.s.	0,9997<br>n.s.
	**Control to *Δnud-2 + him-10+zwl-1(RNAi)***	>0,9999<br>n.s.	0,9993<br>n.s.	>0,9999<br>n.s.	>0,9999<br>n.s.
**Sup. Fig2d**<br>one-way ANOVA with Tukey's multiple comparison (of all 3D conditions)		**-200s to -150s**	**-140s to -100s**	**-90s to -50s**	**-40s to 0s**
	**Control to *zwl-1(RNAi)***	>0,9999<br>n.s.	>0,9999<br>n.s.	>0,9999<br>n.s.	0,9618<br>n.s.
	**Control to *klp-19(RNAi)***	0,9986<br>n.s.	0,0003<br>***	0,6092<br>n.s.	0,1834<br>n.s.
	**Control to *Δnud-2***	0,9980<br>n.s.	0,0192<br>*	0,6045<br>n.s.	0,992<br>n.s.
	**Control to *Δnud-2 + zwl-1(RNAi)***	0,0130<br>*	0,2608<br>n.s.	0,3912<br>n.s.	0,0087<br>**
	**Control to *Δnud-2 + him-10+zwl-1(RNAi)***	<0,0001<br>****	<0,0001<br>****	<0,0001<br>****	<0,0001<br>****
**Sup. Fig5a**<br>one-way ANOVA with Tukey's multiple comparison	**Control to *HIM-10::AID* + auxin + *him-10(RNAi)***	**<0.0001<br>******			
	**Control to *Δska-1***	**0,9026<br>n.s.**			
	HIM-10::AID* + auxin + *him-10(RNAi)* to *Δska-1	**0,0002<br>*****			
**Sup. Fig5e**<br>one-way ANOVA with Tukey's		**-200s to -150s**	**-140s to -100s**	**-90s to -50s**	**-40s to 0s**
	**Control to *Δska-1***	>0,9999<br>n.s.	>0,9999<br>n.s.	0,9941<br>n.s.	0,0972<br>n.s.

multiple comparison (of all 3D conditions)	**Control to NDC-80 *Δtail + ndc-80(RNAi)***	>0,9999<br>n.s.	>0,9999<br>n.s.	0,9998<br>n.s.	0,0058<br>**
	**Control to NDC-80 CH* + *ndc-80(RNAi)***	>0,9999<br>n.s.	>0,9999<br>n.s.	>0,9999<br>n.s.	>0,9999<br>n.s.
**Sup. Fig5f**<br>one-way ANOVA with Tukey's multiple comparison (of all 3D conditions)		**-200s to -150s**	**-140s to -100s**	**-90s to -50s**	**-40s to 0s**
	**Control to *Δska-1***	0,9802<br>n.s.	0,9982<br>n.s.	0,3504<br>n.s.	<0,0001<br>****
	**Control to NDC-80 *Δtail + ndc-80(RNAi)***	<0,0001<br>****	<0,0001<br>****	0,0042<br>**	0,2627<br>n.s.
	**Control to NDC-80 CH* + *ndc-80(RNAi)***	<0,0001<br>****	<0,0001<br>****	<0,0001<br>****	<0,0001<br>****
**Sup. Fig6h**<br>one-way ANOVA with Tukey's multiple comparison (of all 3D conditions)		**-200s to -150s**	**-140s to -100s**	**-90s to -50s**	**-40s to 0s**
	**Control to KNL-1 *Δ85-505 + knl-1(RNAi)***	0,0110<br>*	0,9938<br>n.s.	0,9999<br>n.s.	>0,9999<br>n.s.
**Sup. Fig6i**<br>one-way ANOVA with Tukey's multiple comparison (of all 3D conditions)		**-200s to -150s**	**-140s to -100s**	**-90s to -50s**	**-40s to 0s**
	**Control to KNL-1 *Δ85-505 + knl-1(RNAi)***	0,9995<br>n.s.	0,876<br>n.s.	0,987<br>n.s.	0,9997<br>n.s.
**Sup. Fig7a**<br>one-way ANOVA with Tukey's multiple comparison	**Normal segregators vs laggers at anaphase onset**	<0,0001<br>****			
	**Normal segregators vs cossegregators at anaphase onset**	<0,0001<br>****			
**Sup. Fig7b**<br>t-test with Welch's correction, two-sided	**Correctly vs non correctly segregated chromosomes at anaphase onset**	<0,0001<br>****			

SUPPLEMENTARY REFERENCES

1. Maton, G. *et al.* Kinetochore components are required for central spindle assembly. *Nat Cell Biol* **17**, 697-705 (2015).
2. Gassmann, R. *et al.* A new mechanism controlling kinetochore-microtubule interactions revealed by comparison of two dynein-targeting components: SPDL-1 and the Rod/Zwilch/Zw10 complex. *Genes Dev* **22**, 2385-2399 (2008).
3. Dumont, J., Oegema, K. & Desai, A. A kinetochore-independent mechanism drives anaphase chromosome separation during acentrosomal meiosis. *Nature cell biology* **12**, 894-901 (2010).

4. Edwards, F., Maton, G., Gareil, N., Canman, J.C. & Dumont, J. BUB-1 promotes amphitelic chromosome biorientation via multiple activities at the kinetochore. *Elife* **7** (2018).

5. Cheerambathur, D.K. *et al.* Dephosphorylation of the Ndc80 Tail Stabilizes Kinetochore-Microtubule Attachments via the Ska Complex. *Dev Cell* **41**, 424-437 e424 (2017).
