# Supplementary material for: Kinetochore component function in C. elegans oocytes revealed by 4D tracking of holocentric chromosomes
Source: Nat Commun. 2023 Jul 7;14:4032. doi: 10.1038/s41467-023-39702-z (PMC10329006; doi:10.1038/s41467-023-39702-z)
Supplement: Supplementary file 2 — Reporting Summary [file 41467_2023_39702_MOESM2_ESM.pdf]

nature portfolio

Corresponding author(s): Julien DUMONT

Last updated by author(s): Jun 1, 2023

Reporting Summary

Nature Portfolio wishes to improve the reproducibility of the work that we publish. This form provides structure for consistency and transparency in reporting. For further information on Nature Portfolio policies, see our Editorial Policies and the Editorial Policy Checklist.

Statistics

For all statistical analyses, confirm that the following items are present in the figure legend, table legend, main text, or Methods section.

n/a	Confirmed	
☐	☒	The exact sample size (*n*) for each experimental group/condition, given as a discrete number and unit of measurement
☐	☒	A statement on whether measurements were taken from distinct samples or whether the same sample was measured repeatedly
☐	☒	The statistical test(s) used AND whether they are one- or two-sided<br>*Only common tests should be described solely by name; describe more complex techniques in the Methods section.*
☐	☒	A description of all covariates tested
☐	☒	A description of any assumptions or corrections, such as tests of normality and adjustment for multiple comparisons
☐	☒	A full description of the statistical parameters including central tendency (e.g. means) or other basic estimates (e.g. regression coefficient) AND variation (e.g. standard deviation) or associated estimates of uncertainty (e.g. confidence intervals)
☐	☒	For null hypothesis testing, the test statistic (e.g. *F*, *t*, *r*) with confidence intervals, effect sizes, degrees of freedom and *P* value noted<br>*Give P values as exact values whenever suitable.*
☒	☐	For Bayesian analysis, information on the choice of priors and Markov chain Monte Carlo settings
☒	☐	For hierarchical and complex designs, identification of the appropriate level for tests and full reporting of outcomes
☒	☐	Estimates of effect sizes (e.g. Cohen's *d*, Pearson's *r*), indicating how they were calculated

Our web collection on statistics for biologists contains articles on many of the points above.

Software and code

Policy information about availability of computer code

Data collection	All microscopic data collection were performed using the Metamorph 7 software (Molecular Devices).
Data analysis	Image analysis was performed using the Fiji (ver 2.9.0/1.54d) and Imaris 9 (Oxford Instruments) software.<br>GraphPad Prism 8 was used to generate all graphs and statistics (mentioned in the figure legends), except for color-coded graphs of chromosome orientation, alignment and axial compaction, which were generated by a custom-made python script available upon request. This script is used to plot chromosome orientation, alignment and compaction values of each chromosome every 10 s, and to color-code the proportion of chromosome angles, distances from the spindle equator and distances from the spindle long axis below the thresholds defined previously, at each time points.

For manuscripts utilizing custom algorithms or software that are central to the research but not yet described in published literature, software must be made available to editors and reviewers. We strongly encourage code deposition in a community repository (e.g. GitHub). See the Nature Portfolio guidelines for submitting code & software for further information.

Data

Policy information about availability of data

All manuscripts must include a data availability statement. This statement should provide the following information, where applicable:

- Accession codes, unique identifiers, or web links for publicly available datasets
- A description of any restrictions on data availability
- For clinical datasets or third party data, please ensure that the statement adheres to our policy

All data supporting the findings of this study are available within the paper and its Supplementary Information. Source data for each figure is provided as a separate 'Source Data' Excel file.

Research involving human participants, their data, or biological material

Policy information about studies with human participants or human data. See also policy information about sex, gender (identity/presentation), and sexual orientation and race, ethnicity and racism.

Reporting on sex and gender	Not applicable
Reporting on race, ethnicity, or other socially relevant groupings	Not applicable
Population characteristics	Not applicable
Recruitment	Not applicable
Ethics oversight	Not applicable

Note that full information on the approval of the study protocol must also be provided in the manuscript.

Field-specific reporting

Please select the one below that is the best fit for your research. If you are not sure, read the appropriate sections before making your selection.

☒ Life sciences ☐ Behavioural & social sciences ☐ Ecological, evolutionary & environmental sciences

For a reference copy of the document with all sections, see nature.com/documents/nr-reporting-summary-flat.pdf

Life sciences study design

All studies must disclose on these points even when the disclosure is negative.

Sample size	We followed standard protocols used in the field to choose the sample size. Nevertheless, the sample size we used in this study is always above the average considered sufficient for each specific experimental setting in similar studies doi: 10.7554/eLife.82579; doi: 10.7554/eLife.40690; doi: 10.1038/s41467-017-01539-8
Data exclusions	We did not exclude any data.
Replication	The findings in this study were highly reproducible. As each oocyte is dissected manually and filmed individually, each movie can be considered as an independent biological replicate. The number of biological replicates is thus equal or above 10, collected from 3 independent experiments for every condition tested.
Randomization	For each experiment, the allocation of worms to each group is determined by the experimental treatment they are subjected to. Within each group, worms were then randomly selected from plates containing between 100 and 300 worms.
Blinding	Blinding was not relevant for this study as different experimental conditions, which require specific preparations, are compared. The investigator must thus be aware of the condition being analyzed.

Reporting for specific materials, systems and methods

We require information from authors about some types of materials, experimental systems and methods used in many studies. Here, indicate whether each material, system or method listed is relevant to your study. If you are not sure if a list item applies to your research, read the appropriate section before selecting a response.

Materials & experimental systems

n/a	Involved in the study
☒	☐ Antibodies
☒	☐ Eukaryotic cell lines
☒	☐ Palaeontology and archaeology
☐	☒ Animals and other organisms
☒	☐ Clinical data
☒	☐ Dual use research of concern
☒	☐ Plants

Methods

n/a	Involved in the study
☒	☐ ChIP-seq
☒	☐ Flow cytometry
☒	☐ MRI-based neuroimaging

Animals and other research organisms

Policy information about studies involving animals; ARRIVE guidelines recommended for reporting animal research, and Sex and Gender in Research

Laboratory animals	All the strains used in this study are derived from the ancestral wild-type Caenorhabditis elegans N2 nematode strain. Analyzed nematodes were all 72-96-hours old adults. The strain list with complete genotypes is provided in Supplementary Table 1.
Wild animals	The study did not involve wild animals.
Reporting on sex	Only hermaphrodite worms were used in this study because we studied oocyte meiosis.
Field-collected samples	The study did not involve samples collected from the field.
Ethics oversight	No ethical approval was required for this work performed exclusively on the nematode C. elegans.

Note that full information on the approval of the study protocol must also be provided in the manuscript.
