# Supplementary material for: Kinetochore component function in C. elegans oocytes revealed by 4D tracking of holocentric chromosomes
Source: Nat Commun. 2023 Jul 7;14:4032. doi: 10.1038/s41467-023-39702-z (PMC10329006; doi:10.1038/s41467-023-39702-z)
Supplement: Supplementary file 4 — Description of additional supplementary files [file 41467_2023_39702_MOESM4_ESM.pdf]

Description of additional supplementary files

File name: Supplementary Movie 1

Description: 4D *in utero* live imaging of an oocyte undergoing the first meiotic division.

File name: Supplementary Movie 2

Description: 4D *ex utero* live imaging of an oocyte undergoing the first meiotic division.

File name: Supplementary Movie 3

Description: 4D *ex utero* live imaging of an oocyte undergoing the first meiotic division after

knl-1(RNAi) observed through three different angles.
